# Detecting parallel bursts in *in silico *generated parallel spike train data

**DOI:** 10.1186/1471-2202-16-S1-P134

**Published:** 2015-12-18

**Authors:** Christian Braune, Rudolf Kruse

**Affiliations:** 1 Institute for Knowledge Engineering and Language Processing, Otto von Guericke University, Magdeburg, Germany

## Introduction

Neurons process stimuli as joint groups [1]. With multi-electrode arrays being capable of recording hundreds of channels in parallel the need for computational methods arises to efficiently find hints for such groups in the recorded data. Enumerating all possible subsets of neurons becomes quickly unfeasible if not virtually impossible to do. Therefore, we developed methods for efficiently finding so-called assemblies of synchronously firing neurons in spike train data [2,3]. However, these methods only consider nearly synchronous single activations of neurons and ignore the non-stationary firing rates. It has been shown that the bursting behavior of neurons is a different mode of communication between neurons and has to be considered in the analysis as well [4,5].

## Method

Our method builds upon a previously released algorithm that was intended to find synchronously activated neurons in parallel spike train data. This method uses dynamically placed windows, centered on each single spike, to detect episodes of increased synchrony among the spike trains. By calculating the amount of overlap between a single spike train and the complete set of spike trains new features can be generated. These features allow to identify groups of neurons that show an increased amount of synchronous activations compared to what would be expected under the assumption of independence. By allowing the algorithm to utilize information obtained from the burst detection process (e.g. [6-10]) we can efficiently and effectively identify those groups of neurons that show increased synchronous bursting behavior.

## Conclusion

Using artificially generated data we are able to test our method on a multitude of data sets for which we actually know the true assembly structure. The spike trains are generated in such a way, that their statistical properties match those of *in vitro *recordings of embryonal cortical slices, i.e. the inter-burst interval and intra-burst inter spike interval distributions match. With this setup we test our algorithm on different assembly numbers and sizes. We are then able to distinguish between non-related and related neurons as well as to separate the related ones into different assemblies.
